# Geometric Bounds for Low Steklov Eigenvalues of Finite Volume Hyperbolic Surfaces

**DOI:** 10.1007/s12220-025-01990-w

**Published:** 2025-04-04

**Authors:** Asma Hassannezhad, Antoine Métras, Hélène Perrin

**Affiliations:** 1 School of Mathematics, University of Bristol, Fry Building, Woodland Road, Bristol, BS8 1UG UK; 2 Institut de Mathématiques, Université de Neuchâtel, Rue Emile-Argand 11, 2000 Neuchâtel, Suisse

**Keywords:** Steklov eigenvalues, Hyperbolic surfaces, Eigenvalue lower bounds, 35P15, 58C40

## Abstract

We obtain geometric lower bounds for the low Steklov eigenvalues of finite-volume hyperbolic surfaces with geodesic boundary. The bounds we obtain depend on the length of a shortest multi-geodesic disconnecting the surfaces into connected components each containing a boundary component and the rate of dependency on it is sharp. Our result also identifies situations when the bound is independent of the length of this multi-geodesic. The bounds also hold when the Gaussian curvature is bounded between two negative constants and can be viewed as a counterpart of the well-known Schoen-Wolpert-Yau inequality for Laplace eigenvalues. The proof is based on analysing the behaviour of the corresponding Steklov eigenfunction on an adapted version of thick–thin decomposition for hyperbolic surfaces with geodesic boundary. Our results extend and improve the previously known result in the compact case obtained by a different method.

## Introduction

Let $\Sigma$ be a connected finite volume hyperbolic surface with geodesic boundary, and let $b\ge 1$ denote the number of boundary components. We consider the Dirichlet-to-Neumann map ${{\,\mathrm{\mathscr {D}}\,}}$

$$\begin{aligned} {{\,\mathrm{\mathscr {D}}\,}}: C^\infty (\partial \Sigma )\rightarrow &   C^\infty (\partial \Sigma ) \\ f\mapsto &   \partial _\nu \tilde{f}, \end{aligned}$$

where $\tilde{f}$ is the harmonic extension of *f* to $\Sigma$, and $\nu$ is the outward unit normal vector field along $\partial \Sigma$. By the standard spectral theory for self-adjoint operators, we have that its spectrum is discrete and each eigenvalue has finite multiplicity, see Sect. 2. Let $0 = \sigma _0(\Sigma ) < \sigma _1(\Sigma ) \le \cdots \le \sigma _k(\Sigma ) \le \cdots \nearrow \infty$ be the sequence of its eigenvalue, also called the Steklov eigenvalues. The focus of this paper is on the study of geometric bounds for $\sigma _k(\Sigma )$ when $1 \le k \le b-1$ and $b > 1$.

Note that a lower bound for $\sigma _b(\Sigma )$ can be easily obtained by using the collar theorem and comparing $\sigma _b(\Sigma )$ with the *b*-th mixed Steklov-Neumann eigenvalue of a domain composed of a union of disjoint half-collars about boundary geodesics, and the lower bound will depend only on the length of the boundary (see e.g. [31, Lemma 3]). Hence, the study of the spectral gap and bounds on Steklov eigenvalues becomes an intriguing question when $k < b$. We can refer to them as the *low* Steklov eigenvalues.

Lower bounds for the spectral gap of the Steklov problem on a compact Riemannian manifold with boundary have been studied by José Escobar [16, 17], and later by Pierre Jammes [24] where he obtained a Cheeger-type lower bound, see also [23]. The question of obtaining more explicit geometric bounds for low Steklov eigenvalues has been recently studied in [1, 22, 30, 31].

On a compact hyperbolic surface $\Sigma$, Hélène Perrin [31] obtained a geometric lower bound on a modified version of Cheeger-Jammes’ constant in terms of the length of the shortest multi-geodesic separating $\Sigma$ into $k+1$ connected components, each of them containing at least one boundary component and showed that it is of great relevance in the estimate of low Steklov eigenvalues, in particular, she obtained lower and upper bounds for low eigenvalues of $\Sigma$ in terms of the length of this multi-geodesic. Let us state her result more precisely.

For a given hyperbolic surface $\Sigma$, let ${\mathcal {C}}_k$ be the set of multi-geodesics which consist of a union of disjoint simple closed geodesics, not intersecting $\partial \Sigma$, and dividing $\Sigma$ into $k+1$ connected components, each containing at least one connected component of $\partial \Sigma$. We define

1 $$\begin{aligned} \ell _k := \inf _{{\varvec{c}}\in {\mathcal {C}}_k} |{\varvec{c}}|, \end{aligned}$$

where $|{\varvec{c}}|$ is the length of the multi-geodesic ${\varvec{c}}$. When ${\mathcal {C}}_k=\emptyset$, we set $\ell _k=\infty$.

For a compact hyperbolic surface $\Sigma$ of genus *g* with *b* geodesic boundary components of length $\le 2{{\,\textrm{arsinh}\,}}(1)$, the result in [31] states that, assuming that $g\not = 0$ or $b> 3$, there exists a constant $C_1$, depending only on *b* and on *g*, and a universal constant $C_2$ such that for $1\le k<\lceil \frac{b}{2}\rceil$ we have

2 $$\begin{aligned} C_1\ell _k^2\le \sigma _k\le C_2 \frac{\ell _k}{{\alpha }}, \end{aligned}$$

where $\alpha$ is the minimum length of geodesic boundary components. The inequality also holds for $\lceil \frac{b}{2}\rceil \le k <b$, provided that ${\mathcal {C}}_k\ne \emptyset$ and $\ell _k$ is bounded above in terms of *g* and *b*.

This result can be viewed as a counterpart of a result by Schoen, Wolpert and Yau [34] for Laplace eigenvalues of a closed hyperbolic surface $\Sigma$. They showed that for $1 \le k \le 2g-3$, $\lambda _k$ is bounded above and below by positive constants (depending only on *g* and *k*) times the length of the shortest multi-geodesic dividing $\Sigma$ into $k + 1$ connected components. There have been several studies on extending the Schoen-Wolpert-Yau inequality to noncompact surfaces and investigating the asymptotic behavior as the length of the multi-geodesic tends to zero in [6, 7, 13–15, 20].

In this article, we improve the power of $\ell _k$ in the lower bound of (2) to achieve the optimal power as in the Schoen-Wolpert-Yau inequality. Additionally, we generalise this lower bound by removing the upper bound on the maximum length of boundary components, obtain a lower bound for all $k<b$, and state the result in the context of noncompact finite volume hyperbolic surface.

### Theorem 1.1

Let $\Sigma$ be a finite volume hyperbolic surface with $b \ge 1$ geodesic boundary components. Let $\chi ,g,p$ denote the Euler number of $\Sigma$, the genus and the number of cusps respectively, and let $\beta$ be the maximum length of the boundary components. We define

$$\begin{aligned} K := {\left\{ \begin{array}{ll} b - 1 &  \text {if} \; (g \ge 1 \, or \, p \ge 2) \, and \, b \, \ge 1 , \\ b - 2 &  \text {if} \; g = 0, p = 1 \, and \, b \, \ge 2, \\ b - 3 &  \text {if }\; g = 0, p = 0 \, and \, b \, \ge 3. \end{array}\right. } \end{aligned}$$

Then there exists a positive universal constant *C* such that

$$\begin{aligned} \sigma _k(\Sigma ) \ge \frac{C}{b|\chi |^3} \min \left\{ \frac{1}{(1+\beta )^2e^\beta },\frac{\ell _k}{\beta }\right\} ,\qquad 0<k\le K, \end{aligned}$$

and

$$\begin{aligned} \sigma _{K+1}(\Sigma ) \ge \frac{C}{b\chi ^2(1+\beta )^2e^\beta }. \end{aligned}$$

As a consequence of a topological lemma (see Lemma 3.1), we show that $\ell _k<\infty$ for every $1\le k \le K$. In particular, since there exists a surface for which $\ell _k$ can be arbitrary small, Theorem 1.1 shows that there is always a spectral gap between $\sigma _{K+1}$ and $\sigma _K$ when $\ell _K\rightarrow 0$.

By combining Theorem 1.1 with the classical upper bound for Steklov eigenvalues of compact surfaces given in [9, 21], which can be readily extended to the context of finite volume surfaces, and with the upper bound in [31] stated in (2) above (see Remark 3.7), we have that there exist positive constants $C_3=C_3(\chi ,\beta )$, $C_4=C_4(\chi ,\alpha )$, and $C_5=C_5(\chi ,\beta )$ where $\alpha$ is the minimum length of the boundary components, such that

$$\begin{aligned} C_3 \min \{1,{\ell _k}\}\le \sigma _k(\Sigma ) \le C_4 \min \{1,{\ell _k}\},\qquad 1\le k\le K \end{aligned}$$

and

$$\begin{aligned} C_3\le \sigma _{K+1}(\Sigma ) \le C_5. \end{aligned}$$

We want to highlight here some special cases. With the assumption that $\beta \le 2{{\,\textrm{arsinh}\,}}(1)$, by combining our result with Theorem 3 and Lemma 3 of [31], we have

3 $$\begin{aligned} \frac{C_6}{b |\chi |^3}\min \left\{ 1,\frac{\ell _k}{\beta } \right\} \le \sigma _k(\Sigma ) \le C_7\min \left\{ 1, \frac{\ell _k}{\alpha }\right\} ,\qquad 1\le k\le K, \end{aligned}$$

where $C_6$ and $C_7$ are universal constants.

Assume that $\ell _k$ is bounded above in terms of a constant depending only on $\chi$. It is the case for example when $k<\min \{\lceil \frac{b}{2}\rceil , K+1\}$ as shown in [31]. Then there exist positive constants $C_8(\chi , \beta )$ and $C_9(\beta )$ such that

$$C_8{\ell _k} \le \sigma _k\le C_9\frac{\ell _k}{ \alpha },$$

and $C_8$ and $C_9$ can be independent of $\beta$ when $\beta \le 2{{\,\textrm{arsinh}\,}}(1)$; this recovers an improved version of (2) with an optimal dependency on $\ell _k$. Figure 5 illustrates an example for which $\ell _k$ can be arbitrarily large for $k=\lceil \frac{b}{2}\rceil$. Hence, the above bound cannot hold for $k\ge \min \{\lceil \frac{b}{2}\rceil , K+1\}$ in general.

When $\ell _k$ tends to zero, the combination of Theorem 1.1 and the upper bound in [31] as stated in (2) (see Remark 3.7) implies that

4 $$\begin{aligned} \frac{C}{b|\chi |^3\beta }\le \liminf _{\ell _k\rightarrow 0}\frac{\sigma _k}{\ell _k}\le \limsup _{\ell _k\rightarrow 0}\frac{\sigma _k}{\ell _k}\le \frac{C_2}{\alpha },\qquad 1\le k\le K, \end{aligned}$$

where *C* and $C_2$ are positive universal constants as mentioned above. In particular, when $\beta =\alpha$ or $\alpha$ is a constant multiple of $\beta$, it shows the optimality of the power of $\beta$. In general, when $\chi$ or $\beta /\alpha$ is large, there will be a big gap between the upper and lower bound in inequality (4). The study of the asymptotic of $\frac{\sigma _k}{\ell _k}$ as $\ell _k\rightarrow 0$ is an intriguing question. We refer to [6, 10, 12, 20] for related studies for the Laplace eigenvalues.

It is also very interesting to investigate the optimality of the dependency of the lower bound on $\chi$. The power of $|\chi |$ in our lower bound for $\sigma _1$ obtained in Proposition 3.3 is $-2$. From [1, Example 5.1], we know that there exists a sequence of hyperbolic surfaces for which $\sigma _{b-1}$ decays at rate $\frac{1}{|\chi |}$. It remains open whether the optimal power of $|\chi |$ is $-1$. In the case of the Laplacian, Wu and Xue [36] showed that the optimal power of $|\chi |$ in the lower bound for the first non-zero Laplace eigenvalue of a closed hyperbolic surface is $-2$.

The proof of Theorem 1.1 uses a different approach that the one used in [31]. It is inspired by Dodziuk-Randol’s proof of the Schoen-Wolpert-Yau inequality in [15], see also [13, 14]. Inspired by their approach, we analyse the behaviour of Steklov eigenfunction on an adapted version of the thick–thin decomposition of a hyperbolic surface. A similar adaptation is also used in [1] to obtain a geometric lower bound for the spectral gap in pinched negatively curved manifolds of dimension at least 3. However, the situation differs in dimension 2; unlike higher dimensions, the *thick* part is not connected, presenting its own challenge.

Since the Steklov eigenvalues are invariant under any conformal change in the interior, Theorem 1.1 holds true for any Riemannian surface $\Sigma$ that is conformally equivalent to a hyperbolic surface with geodesic boundaries, with a conformal factor equal to 1 along the boundary. Let $(\Sigma ,h)$ be conformal to a hyperbolic surface $(\Sigma ,{\bar{h}})$ with geodesic boundary and the conformal factor *f*, where $h=f\,{\bar{h}}$, satisfies $A^{-1} \le f|_{\partial \Sigma }\le A$ for some constant $A\ge 1$. Then using the variational characterisation of the Steklov eigenvalues, we get

$$\begin{aligned} A^{{-1/2}}\, \sigma _k(\Sigma ,{\bar{h}})\le \sigma _k(\Sigma ,h)\le A^{{1/2}}\sigma _k(\Sigma ,{\bar{h}}). \end{aligned}$$

When $(\Sigma , h)$ is a compact Riemannian surface with Gaussian curvature in the interval $[-1, -\kappa ]$ for some $\kappa > 0$, we show that it is conformal to a hyperbolic surface $(\Sigma , {\bar{h}})$ with geodesic boundary. Moreover, the conformal factor *f* satisfies $1 \le f \le \kappa ^{-1}$, ensuring that all results above remain valid, with ${\kappa }$ appearing as a multiplicative factor in the lower bound. For non-compact finite-volume surfaces with Gaussian curvature within the same range, similar bounds still hold. For further details, see Theorem 3.8.

In general, we can ask whether one can conformally deform a surface with boundary to obtain a hyperbolic surface with geodesic boundary while the conformal factor remains bounded. Uniformisation theorems for surfaces with boundary are studied in [4, 5, 29, 33]. In particular, it is known that for a compact Riemannian surface $(\Sigma ,h)$ with boundary, when the integral of the geodesic curvature along $\partial \Sigma$ is non-negative, there exists a unique hyperbolic metric ${\bar{h}}=f h$ in the conformal class of *h* such that the boundary of $(\Sigma ,{\bar{h}})$ is geodesic. The metric ${\bar{h}}$ is called a *uniform metric*. However, the resulting surface may not be compact.

**Fig. 1:**
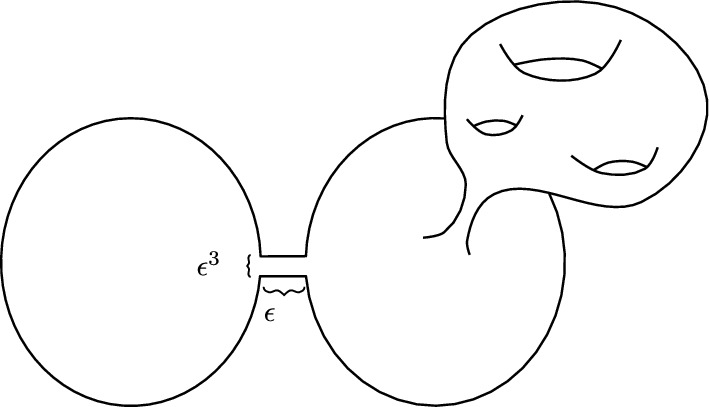
$\Sigma _\epsilon$ is obtained as a union of two discs connected by a thin neck of length $\epsilon$ and width $\epsilon ^3$, and removing a small disc around the centre of one of the discs, then performing a connected sum with a surface of genus at least 1

We can construct examples of a sequence of Riemannian surfaces $\Sigma _\epsilon$ with $\chi (\Sigma _\epsilon ) < 0$, such that for any given $k \ge 1$, $\lim _{\epsilon \rightarrow 0} \sigma _k(\Sigma _\epsilon ) = 0$. This sequence can be constructed by slightly modifying the example given in [18, Sect. 2.2], as illustrated in Fig. 1. This demonstrates that for $\epsilon$ small enough, $\Sigma _\epsilon$ cannot be conformally equivalent to a hyperbolic surface with a conformal factor equal to 1 on the boundary. Moreover, by slightly modifying the example above, we can assume the geodesic curvature along $\partial \Sigma$ is non-negative. Hence, the conformal factor of the uniform metric along the boundary cannot remain uniformly bounded, and the answer to the question above is negative.

The paper is organised as follows. In Sect. 2, we cover some preliminaries, including the definition of an adapted version of the thick–thin decomposition for hyperbolic surfaces with geodesic boundary, and the result of Dodziuk and Randol [15] on the behaviour of eigenfunctions in the thick and thin parts. Section 3 is devoted to the proof of the main result. We first present a topological lemma demonstrating when $\ell _k$ is achieved. Then we prove Theorem 1.1 for $k=1$ and show that the main theorem can be derived from this case.

## Preliminaries

Throughout the paper, we assume that $\Sigma$ is a finite volume connected hyperbolic surface with nonempty geodesic boundary unless otherwise stated. We say that $\Sigma$ is of signature (*g*, *b*; *p*) if it has genus *g*, *b* geodesic boundary components, and *p* cusps.

### Steklov Problem

When $\Sigma$ is compact, i.e. is of signature (*g*, *b*; 0), the Dirichlet-to-Neumann map ${{\,\mathrm{\mathscr {D}}\,}}$

$$\begin{aligned} {{\,\mathrm{\mathscr {D}}\,}}: C^\infty (\partial \Sigma )\rightarrow &   C^\infty (\partial \Sigma ) \\ f\mapsto &   \partial _\nu \tilde{f}, \end{aligned}$$

where $\tilde{f}$ is the harmonic extension of *f* to $\Sigma$, and $\nu$ is the outward unit normal vector field along $\partial \Sigma$, is a self-adjoint first-order elliptic pseudo-differential operator and its spectrum consists of a discrete sequence of non-negative real numbers with the only accumulation point at infinity, see e.g. [27].

The Dirichlet-to-Neumann operator on non-compact geometrically finite manifolds has been recently studied in [32]. However, in our setting, we explain that the discreteness of its spectrum is a consequence of classical theory.

Given a finite volume hyperbolic surface $\Sigma$, let $\mathcal {D}\Sigma$ denote the double of $\Sigma$ along its geodesic boundaries. It is a complete finite volume hyperbolic surface. We first briefly recall the spectral theory of the Laplace-Beltrami operator on a finite volume noncompact hyperbolic surface. It is well-known that $\Delta$ is essentially self-adjoint and has a unique Friedrich extension. Its spectrum consists of a sequence of eigenvalues $0=\lambda _0<\lambda _1\le \lambda _2\le \cdots$ and continuous spectrum $[\frac{1}{4},\infty )$ (see e.g. [28]). There are finitely many eigenvalues in interval $[0,\frac{1}{4})$. The multiplicity of 0 is 1 and corresponds to the constant functions.

Let us consider the Dirichlet Laplacian on $\Sigma$. Then we immediately get that the bottom of the Dirichlet spectrum

$$\begin{aligned} \lambda _0^D(\Sigma )=\inf _{0\ne f\in H^1_0(\Sigma )}\frac{\int _\Sigma |\nabla f|^2}{\int _\Sigma f^2} \end{aligned}$$

is strictly positive because $\lambda _0^D(\Sigma )\ge \min \{1/4,\lambda _1(\mathcal {D}\Sigma )\}$. It implies that for any $f\in C^\infty (\partial \Sigma )$, there is a unique square integrable harmonic extension $\tilde{f}$ to $\Sigma$. Hence, the Dirichlet-to-Neumann map ${{\,\mathrm{\mathscr {D}}\,}}$ is well defined. It is symmetric and positive. We consider its Friedrich extension, also denoted by ${{\,\mathrm{\mathscr {D}}\,}}$. The map ${{\,\mathrm{\mathscr {D}}\,}}$ is a first-order elliptic operator and its spectrum consists of a discrete sequence of non-negative real numbers with the only accumulation point at infinity. The discreteness of the spectrum follows from the compactness of the trace operator $T:H^1(\Sigma )\rightarrow L^2(\partial \Sigma )$. The eigenvalues of the Dirichlet-to-Neumann map are the same as the eigenvalues of the Steklov problem:

$${\left\{ \begin{array}{ll} \Delta u = 0 &  \text {in } \Sigma ,\\ \partial _\nu u = \sigma u & \text {on } \partial \Sigma . \end{array}\right. }$$

We enumerate them in increasing order counting their multiplicities:

$$\begin{aligned}0 = \sigma _0 < \sigma _1 \le \sigma _2 \le \cdots \nearrow \infty .\end{aligned}$$

We have the following variational characterisation of the Steklov eigenvalues.

5 $$\begin{aligned} \sigma _k=\inf _{V_{k+1}}\sup _{{0\ne } f\in V_{k+1}}\frac{\int _\Sigma |\nabla f|^2}{\int _{\partial \Sigma } f^2},\end{aligned}$$

where $V_{k+1}$ is a $(k+1)$-dimensional subspace of $H^1(\Sigma )$.

In the subsequent sections, we also consider the mixed Steklov-Dirichlet and mixed Steklov-Neumann eigenvalue problems, where either the Dirichlet or Neumann boundary condition is assumed on a portion of the boundary.

The variational characterization of the Steklov-Neumann and Steklov-Dirichlet eigenvalues is similar to that of the Steklov eigenvalues. The only difference is that the integration in the denominator of the Rayleigh quotient in (5) is restricted to the Steklov part of the boundary. For the Steklov-Dirichlet problem, we should also restrict the functional space to those functions that vanish on the part of the boundary with the Dirichlet condition.

### Thick–Thin Decomposition

We define the thick–thin decomposition of $\Sigma$ as follows. Let $\mathcal {D}\Sigma$ be the double of $\Sigma$ along its totally geodesic boundary. We define $(\mathcal {D}\Sigma )_{\scriptscriptstyle {\text {thin}}}$ to be the subset of $\mathcal {D}\Sigma$ consisting of

(i) the union of collars ${{\,\mathrm{\mathscr {C}}\,}}(\gamma )$ for all simple closed geodesic of length $\le 2{{\,\textrm{arsinh}\,}}(1)$:
  $$\begin{aligned} {{\,\mathrm{\mathscr {C}}\,}}(\gamma )=\left\{ p\in \mathcal {D}\Sigma \,|\, {{\,\textrm{dist}\,}}(p,\gamma )\le {{\,\textrm{w}\,}}(\gamma )\right\} ,\quad \text {where}\quad {{\,\textrm{w}\,}}(\gamma )={{\,\textrm{arsinh}\,}}\left( \frac{1}{\sinh (|\gamma |/2)}\right) \end{aligned}$$
  and $|\gamma |$ denotes the length of $\gamma$. The collar ${{\,\mathrm{\mathscr {C}}\,}}(\gamma )$ is isometric to the warped product $[-{{\,\textrm{w}\,}}(\gamma ),{{\,\textrm{w}\,}}(\gamma )]\times _{\cosh }\mathbb {S}^1_{R}$, where $2\pi R=|\gamma |$. Recall that the warped product $[-{{\,\textrm{w}\,}}(\gamma ),{{\,\textrm{w}\,}}(\gamma )]\times _{\cosh }\mathbb {S}^1_{R}$ is the Riemannian surface $[-{{\,\textrm{w}\,}}(\gamma ),{{\,\textrm{w}\,}}(\gamma )]\times \mathbb {S}^1_{R}$ with Riemannian metric $\, \textrm{d}t^2+\cosh ^2( t) g_{\mathbb {S}^1_{R}}$ where $\, \textrm{d}t^2$ and $g_{\mathbb {S}^1_{R}}$ denote the canonical metrics of $[-{{\,\textrm{w}\,}}(\gamma ),{{\,\textrm{w}\,}}(\gamma )]$ and $\mathbb {S}^1_{R}$;
(ii) a finite collection of cusps ${{\,\mathrm{{\mathscr {K}}}\,}}$ isometric to the warped product $(-\infty , \log 2)\times _f\mathbb {S}^1$, where $f(t)=e^{t}$.

According to the Collar Theorem [8, Theorem 4.4.6], the collars and cusps are mutually disjoint and the injectivity radius of any point in the complement of $(\mathcal {D}\Sigma )_{\scriptscriptstyle {\text {thin}}}$ is strictly bigger than ${{\,\textrm{arsinh}\,}}(1)$. By defining

$$\begin{aligned} (\mathcal {D}\Sigma )_{\scriptscriptstyle {\text {thick}}}:=\{p\in \mathcal {D}\Sigma \,|\, {{\,\textrm{inj}\,}}_p(\mathcal {D}\Sigma )>{{\,\textrm{arsinh}\,}}(1)\}, \end{aligned}$$

we have a covering of $\mathcal {D}\Sigma$ by $(\mathcal {D}\Sigma )_{{\scriptscriptstyle {\text {thick}}}}$ and $(\mathcal {D}\Sigma )_{\scriptscriptstyle {\text {thin}}}$ which we call the *thick–thin decomposition*. Note that $(\mathcal {D}\Sigma )_{{\scriptscriptstyle {\text {thick}}}}$ is always nonempty, and if $(\mathcal {D}\Sigma )_{\scriptscriptstyle {\text {thin}}}\ne \emptyset$, the intersection of the thick and thin parts is nonempty. Indeed, for any point on $p\in \partial {{\,\mathrm{\mathscr {C}}\,}}(\gamma )\subset (\mathcal {D}\Sigma )_{\scriptscriptstyle {\text {thin}}}$, by [8, Theorem 4.1.6]), we have ${{\,\textrm{inj}\,}}_p(\mathcal {D}\Sigma )>{{\,\textrm{arsinh}\,}}(1)$.

We can view $\Sigma$ as a subdomain of $\mathcal {D}\Sigma$ and define its thick–thin decomposition by considering the intersection of $\Sigma$ with the thick and thin parts of $\mathcal {D}\Sigma$. However, we shall need to consider an alternative definition described below.

**Fig. 2:**
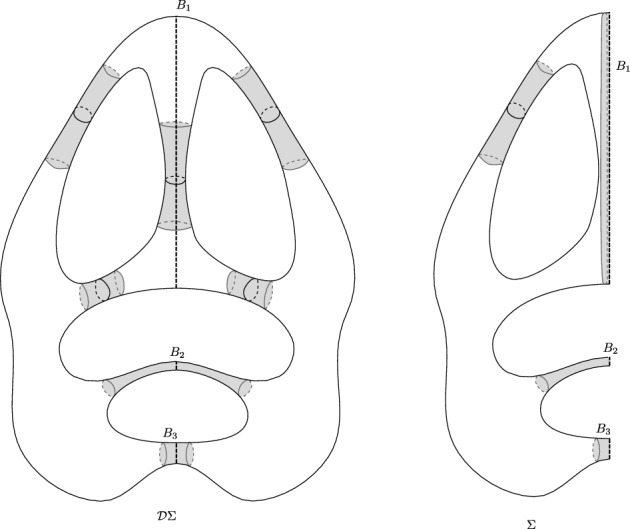
On the left, the grey parts show $(\mathcal {D}\Sigma )_{\scriptscriptstyle {\text {thin}}}$ where $\Sigma$ is a hyperbolic surface with 3 boundary components $B_1, B_2$, and $B_3$, and on the right, the thin part $\Sigma _{\scriptscriptstyle {\text {thin}}}^{{\varepsilon _\circ }}$ of $\Sigma$. Note that since ${\varepsilon _\circ }\le {{\,\textrm{arsinh}\,}}(1)$, some of the original thin tubes are no longer in the thin part. Furthermore, by definition, the half-collar of each boundary component is part of $\Sigma _{\scriptscriptstyle {\text {thin}}}^{{\varepsilon _\circ }}$

We observe that the intersection between $\Sigma$ and a collar ${{\,\mathrm{\mathscr {C}}\,}}(\gamma ){\subset (\mathcal {D}\Sigma )_{\scriptscriptstyle {\text {thin}}}}$ is the collar itself if $\gamma \subset \Sigma$; it is a half-collar if $\gamma$ is one of the boundary components. But if there is at least one geodesic boundary of $\Sigma$ of length $>2{{\,\textrm{arsinh}\,}}(1)$, we may have that $\gamma \cap \Sigma$ is a geodesic arc with endpoints on one or two boundary components (as it happens in Fig. 2 where a geodesic of the decomposition intersect the geodesic boundary). For technical reasons, we want to avoid this situation. Hence, to define the thick–thin decomposition for $\Sigma$, we first modify the definition of the thick–thin decomposition of $\mathcal {D}\Sigma$.

Let $\{B_1,\cdots , B_b\}$ be the boundary components of $\partial \Sigma$ and $\beta =|B_{\max }|=\max _i|B_i|$. We take

6 $$\begin{aligned} {\varepsilon _\circ }={\varepsilon _\circ }(\beta )=\min \left\{ {{{\,\textrm{arsinh}\,}}(1)}, {{\,\textrm{w}\,}}(B_{\max })\right\} ,\end{aligned}$$

and define the ${\varepsilon _\circ }$-*thick–thin decomposition* of $\mathcal {D}\Sigma$ as follows.

$$(\mathcal {D}\Sigma )_{{\scriptscriptstyle {\text {thick}}}}^{\varepsilon _\circ }=\{p\in \mathcal {D}\Sigma \,|\, {{\,\textrm{inj}\,}}_p(\mathcal {D}\Sigma )>{{\varepsilon _\circ }}\},\qquad (\mathcal {D}\Sigma )_{\scriptscriptstyle {\text {thin}}}^{\varepsilon _\circ }=\bigcup _{|\gamma |\le 2{\varepsilon _\circ }}{{\,\mathrm{\mathscr {C}}\,}}(\gamma )\bigcup \left( \bigcup _j {{\,\mathrm{{\mathscr {K}}}\,}}_j\right) .$$

The union of the ${\varepsilon _\circ }$-thick and ${\varepsilon _\circ }$-thin parts cover the whole $\mathcal {D}\Sigma$ because if $p\in \mathcal {D}\Sigma \setminus \mathcal {D}\Sigma _{\scriptscriptstyle {\text {thin}}}^{\varepsilon _\circ }$, either $p\in {{\,\mathrm{\mathscr {C}}\,}}(\gamma )$ for a $\gamma$ with $2{{\,\textrm{arsinh}\,}}(1)\ge |\gamma |\ge 2{\varepsilon _\circ }$ which implies ${{\,\textrm{inj}\,}}_p(\mathcal {D}\Sigma )\ge {\varepsilon _\circ }$, or $p \in \mathcal {D}\Sigma _{{\scriptscriptstyle {\text {thick}}}}$ and ${{\,\textrm{inj}\,}}_p(\mathcal {D}\Sigma )>{{\,\textrm{arsinh}\,}}(1)\ge {\varepsilon _\circ }$. We now define the ${\varepsilon _\circ }$-thick–thin decomposition of $\Sigma$ as follows.

$$ \Sigma _{{\scriptscriptstyle {\text {thick}}}}^{\varepsilon _\circ }:=(\Sigma \cap (\mathcal {D}\Sigma )_{{\scriptscriptstyle {\text {thick}}}}^{\varepsilon _\circ })\setminus (\cup _j{{\,\mathrm{\mathscr {C}}\,}}^+_j)^{\circ },\qquad \Sigma _{\scriptscriptstyle {\text {thin}}}^{\varepsilon _\circ }:=((\mathcal {D}\Sigma )_{\scriptscriptstyle {\text {thin}}}^{\varepsilon _\circ }\cap \Sigma )\cup (\cup _j{{\,\mathrm{\mathscr {C}}\,}}^+_j)),$$

where ${{\,\mathrm{\mathscr {C}}\,}}_j^+$ denote the half-collar around the geodesic boundary $B_j$ and $({{\,\mathrm{\mathscr {C}}\,}}_j^+)^{\circ }$ its interior. We note that for any point $p \in \Sigma _{{\scriptscriptstyle {\text {thick}}}}^{\varepsilon _\circ }$, ${{\,\textrm{inj}\,}}_p(\Sigma )\ge {\varepsilon _\circ }$ because ${{\,\textrm{inj}\,}}_p(\mathcal {D}\Sigma )>{\varepsilon _\circ }$ and ${{\,\textrm{dist}\,}}(p,\partial \Sigma )\ge {\varepsilon _\circ }$. We also note that $\Sigma _{\scriptscriptstyle {\text {thin}}}^{\varepsilon _\circ }$ is a disjoint union of collars, half-collars, and cusps.

We shall see that a key ingredient of the proof is the behaviour of the Steklov eigenfunctions on the half-collars, the thick part and the thin collars, while the presence of the cusps will not be important. We list two key lemmas due to Dodziuk and Randol [13, 15] which provide estimates on the Dirichlet energy of the Steklov eigenfunction on the thick part and on thin collars.

#### Lemma 2.1

([15]) Let ${{\,\mathrm{\mathscr {C}}\,}}(\gamma )\subset (\mathcal {D}\Sigma )_{\scriptscriptstyle {\text {thin}}}$ and $\Gamma _1$ and $\Gamma _2$ be the two boundary components of ${{\,\mathrm{\mathscr {C}}\,}}(\gamma )$. Let *f* be a differentiable function on ${{\,\mathrm{\mathscr {C}}\,}}(\gamma )$ and assume that there exists a positive constant $c>0$ such that

$$\begin{aligned} \min _{x \in \Gamma _1} |f(x) - f(x^*)| \ge c, \end{aligned}$$

where $x^* \in \Gamma _2$ is the reflection of $x \in \Gamma _1$ with respect to $\gamma$. Then

$$\begin{aligned} \int _{{{\,\mathrm{\mathscr {C}}\,}}(\gamma )}|\nabla f|^2 \ge \frac{c^2 |\gamma |}{4}. \end{aligned}$$

The second key lemma shows that the oscillation of a Steklov eigenfunction on the thick part is bounded above by the corresponding Steklov eigenvalue.

#### Lemma 2.2

If $\varphi$ is a $\sigma$-Steklov eigenfunction with $\Vert \varphi \Vert _{L^2(\partial \Sigma )}=1$, then for any *x*, *y* that belongs to a single connected component $\Sigma _0$ of ${\Sigma ^{{\varepsilon _\circ }}_{{\scriptscriptstyle {\text {thick}}}}}$, we have

7 $$\begin{aligned} |\varphi (x)-\varphi (y)|\le c(\beta ) \sqrt{\sigma |\Sigma _0|}, \end{aligned}$$

where $c(\beta )=c\,{\varepsilon _\circ }^{-1}$ for some positive universal constant *c*. Note that $c(\beta )$ depends on $\beta =|B_{\max }|$ only when $\beta \ge 2{{\,\textrm{arsinh}\,}}(1)$, otherwise it is independent of $\beta$.

#### Proof

This result is a consequence of the fact that there exists a positive universal constant $c_1$ such that for any harmonic function $\varphi$ on $\Sigma$ and for any ball ${{\mathcal {B}}}$ centered at a point $x\in \Sigma ^{{\varepsilon _\circ }}_{{\scriptscriptstyle {\text {thick}}}}$ of radius $0<r\le {\varepsilon _\circ }$ we have

8 $$\begin{aligned} \Vert \nabla \varphi \Vert _{\infty ,{{\mathcal {B}}}/2}\le c_1r^{-1}\left( \int _{{\mathcal {B}}}|\nabla \varphi |^2\, \textrm{d}A\right) ^{\frac{1}{2}}, \end{aligned}$$

where ${{\mathcal {B}}}/2$ is the ball concentric with ${{\mathcal {B}}}$ and of half the radius of ${{\mathcal {B}}}$. We refer to [15] and [13, p. 32] for the proof of inequality (8). See also [1, Sect. 4]. The proof of its consequence, inequality (7), can be also found in [1, 15] but for the convenience of the reader, we add the details of the proof here.

Let $\{{{\mathcal {B}}}_j\}_{j=1}^N$, $N\in \mathbb {N}$ be a chain of overlapping balls centered at $x_j\in \Sigma _0 \subset \Sigma ^{\varepsilon _\circ }_{{\scriptscriptstyle {\text {thick}}}}$, and of radius $r_j={\varepsilon _\circ }/2$, connecting *x* and *y* such that $\{{{\mathcal {B}}}_j/2\}_{j=1}^N$ are mutually disjoint. Then *N* can be bounded above by $c_2{\varepsilon _\circ }^{-2}|\Sigma _0|$. We can also assume that each ball $2{{\mathcal {B}}}_j$ intersects at most $c_3$ balls, where $c_2$ and $c_3$ are universal constants. Then

$$\begin{aligned} \sum _j \Vert \nabla \varphi \Vert _{\infty , {{\mathcal {B}}}_j }\le &   c_1{\varepsilon _\circ }^{-1}\sum _{j=1}^N\left( \int _{2{{\mathcal {B}}}_j}|\nabla \varphi |^2\right) ^{1/2} \\\le &   c_1{\varepsilon _\circ }^{-1} \sqrt{N}\left( \sum _{j=1}^N\int _{2{{\mathcal {B}}}_j}|\nabla \varphi |^2\right) ^{1/2}\\\le &   c_4{\varepsilon _\circ }^{-2} \sqrt{|\Sigma _0|}\left( \int _{\Sigma }|\nabla \varphi |^2\right) ^{1/2}\\= &   c_4{\varepsilon _\circ }^{-2} \sqrt{|\Sigma _0|\sigma }, \end{aligned}$$

where $c_4$ is a universal constant depending on $c_1,c_2$, and $c_3$.

Now, let ${\varvec{c}}:[0,1]\rightarrow \Sigma _0$ be a piece-wise geodesic curve connecting *x* and *y*. We choose the partition $0=t_0<t_1< \cdots <t_m=1$ such that ${\varvec{c}}|_{[t_j,t_{j+1}]}$ is a geodesic and ${\varvec{c}}([t_j,t_{j+1}])\subset {{\mathcal {B}}}_{j}$. Hence, the length of ${\varvec{c}}|_{[t_j,t_{j+1}]}$ is bounded above by $2{\varepsilon _\circ }$. We conclude that

9 $$\begin{aligned} |\varphi (x)-\varphi (y)|= &   \left| \int _0^1\frac{d}{dt}\varphi \circ {\varvec{c}}(t)dt\right| \nonumber \\\le &   \sum _{j}\Vert \nabla \varphi \Vert _{\infty , B_j }\int _{t_j}^{t_{j+1}}|{\varvec{c}}'(t)|dt\nonumber \\\le &   2{\varepsilon _\circ }\sum _{j}\Vert \nabla \varphi \Vert _{\infty , B_{j} }\nonumber \\\le &   c {\varepsilon _\circ }^{-1}\sqrt{|\Sigma _0|}\sigma , \end{aligned}$$

where *c* is a universal constant. $\square$

## Proof of the Main Result

Let us recall the definition of $\ell _k:= \inf _{{\varvec{c}}\in {\mathcal {C}}_k} |{\varvec{c}}|,$ where ${\mathcal {C}}_k$ denotes the set of multi-geodesics formed by disjoint simple closed geodesics, dividing $\Sigma$ into $k+1$ connected components, each containing at least one part of $\partial \Sigma$. When ${\mathcal {C}}_k=\emptyset$, we set $\ell _k=\infty$. The following lemma shows when $\ell _k$ will be achieved.

### Lemma 3.1

Let $\Sigma$ be a hyperbolic surface of signature (g, b; p). Then

(i) ${\mathcal {C}}_b=\emptyset$ for any signature. In particular, ${\mathcal {C}}_1=\emptyset$ when $b=1$.
(ii) ${\mathcal {C}}_1 = \emptyset$ when $(g, b; p)=(0,3;0)$ or (0, 2; 1).
(iii) ${\mathcal {C}}_{b-1}\ne \emptyset$ when $(g \ge 1\text {~or~} p\ge 2)$ and $b \ge 2$.
(iv) ${\mathcal {C}}_{b-2} \ne \emptyset$ and ${\mathcal {C}}_{b-1} = \emptyset$ for any surface with signature (0, *b*; 1), $b \ge 3$.
(v) ${\mathcal {C}}_{b - 3}\ne \emptyset$ and ${\mathcal {C}}_{b-2} = \emptyset$ for any surface with signature (0, *b*; 0), $b \ge 4$.

### Proof

**Fig. 3:**
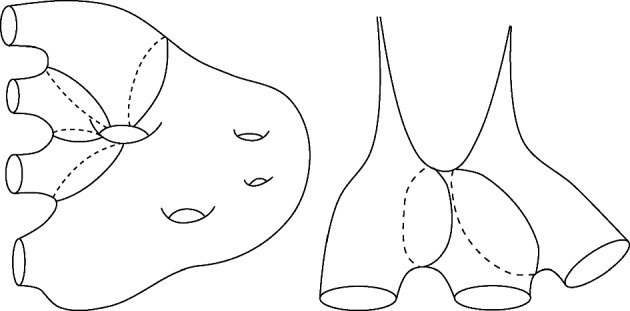
Examples of decomposition when the genus is non-zero or there are at least two cusps

**Fig. 4:**
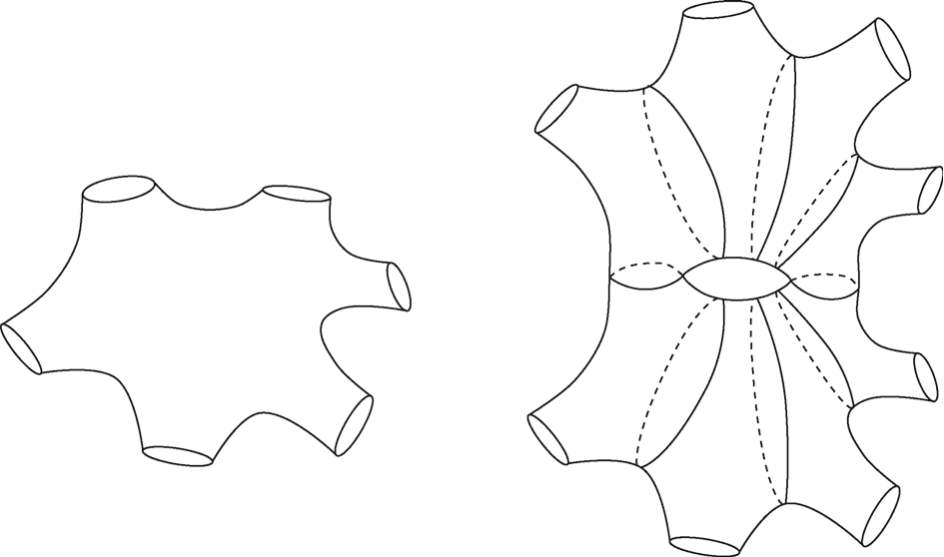
A surface with 6 boundary components and genus 0 and how to obtain 4 disjoint components each containing part of the boundary by considering its double with respect to two of the boundary components

(i) It is clear that ${\mathcal {C}}_b = \emptyset$ as that would require finding a decomposition of $\Sigma$ into $b+1$ components each containing part of the *b* components of the boundary.
(ii) In this case, the surface is either a pair of pants or a surface with two boundary components and one cusp. In either case, one can see that ${\mathcal {C}}_1 = \emptyset$ as there are no geodesic loops (non-homotopic to the boundary components).
(iii) To show that ${\mathcal {C}}_{b-1}$ is non-empty, we proceed as shown in Fig. 3. Note that the curves are not in the same free homotopy class and can be viewed as geodesics. Indeed, if two nontrivial closed curves are disjoint, then the closed geodesics in their respective free homotopy class either coincide as point sets or remain disjoint (see e.g. [8, Chapter 1]).
(iv) [MYAMP (v)] The cases (iv) and (v) are similar in their proof, we first prove case (*v*). Let $B_1$ and $B_2$ be two boundary components and $\mathcal {D}_{B_1,B_2}\Sigma$ be a surface obtained by the doubling of $\Sigma$ along these two boundary components (as illustrated in Fig. 4). Then $\mathcal {D}_{B_1,B_2}\Sigma$ is a surface of signature $(1,2(b-2);0)$. Applying the proof of part (iii), we can obtain a multi-geodesic ${\mathcal {C}}$ decomposing $\mathcal {D}_{B_1, B_2}\Sigma$ into $2b - 4$ components and such that $B_1$ and $B_2$ are part of ${\mathcal {C}}$. This chain when restricted to $\Sigma$ decomposes $\Sigma$ into $b - 2$ components, each having part of the boundary $\partial \Sigma$. Hence ${\mathcal {C}}|_\Sigma \in {\mathcal {C}}_{b-3} \ne \emptyset$. A similar reasoning shows that ${\mathcal {C}}_{b-2} = \emptyset$. For the case (*iv*), the proof is similar but we double along a single boundary component to obtain a surface of signature $(0, 2(b-1); 2)$.

$$\square $$

### Remark 3.2

**Fig. 5:**
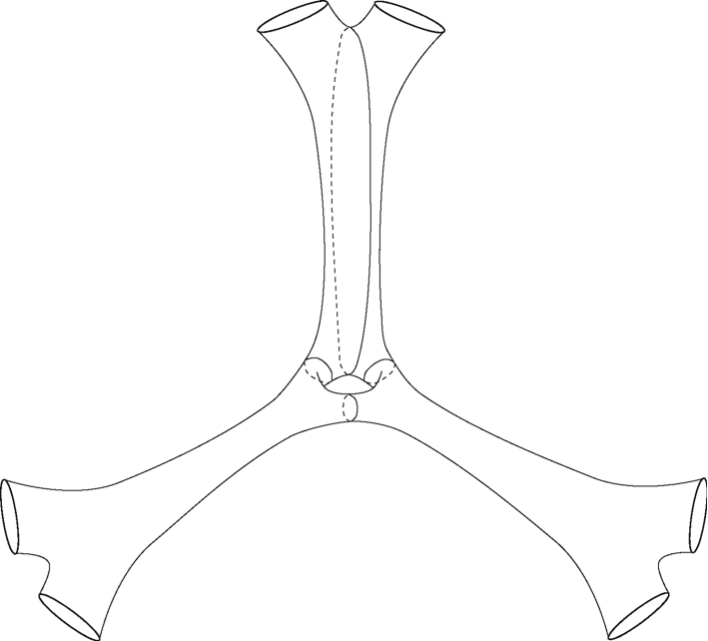
Example of surface with 6 boundary components and a large $\ell _3$

When $\beta < 2 {{\,\textrm{arsinh}\,}}(1)$ and $k < \min \left\{ \lceil \frac{b}{2} \rceil , K+1\right\}$, $\ell _k$ cannot be arbitrarily large. Indeed, as shown in [31], it follows from Bers’ theorem that in this case, $\ell _k$ is bounded in terms of an explicit constant depending only on the genus, number of cusps and number of boundary components. Note that even though the result given in [31] is for compact surfaces, one can easily extend it to allow for cusps by using an appropriate generalisation of Bers’ theorem (see e.g. [8] or [3, Theorem 6.10 and its proof]). On the other hand, for $k \ge \min \{\lceil \frac{b}{2} \rceil , K+1\}$, one can construct surfaces making $\ell _k$ arbitrarily large. For example, Fig. 5 illustrates that one can make $\ell _3$ arbitrarily large while keeping the length of the boundary components constant. This behaviour contrasts with the one observed for the equivalent $\ell _k$ used in the Laplacian eigenvalue problem. In this case, it is always bounded from above by some constant depending on the genus and number of cusps, a consequence of the bound on lengths of pants decomposition [3, 8].

### Proposition 3.3

Let $\Sigma$ be a hyperbolic surface of signature (*g*, *b*; *p*). Let $\beta$ denote the maximum length of the boundary components and $\chi$ the Euler number of $\Sigma$. Then there exists a universal positive constant *c* such that

$$\begin{aligned} \sigma _1(\Sigma ) \ge \frac{c}{b\chi ^2} \min \left\{ \frac{1}{(1+\beta )^2e^\beta },\frac{\ell _1}{\beta }\right\} . \end{aligned}$$

Let us first give an outline of the proof.

*Sketch of the proof* We consider the behaviour of a normalised $\sigma _1$-eigenfunction *f* on ${\varepsilon _\circ }$-thick and thin parts. We first analyse the behaviour of *f* on the $\epsilon$-thin part adjacent to the boundary where it has the largest $L^2$-norm. If *f* is ‘almost’ $L^2$-orthogonal to 1 along that boundary component, then we can modify it and use it as a test function to compare $\sigma _1$ and the first non-zero Steklov-Neumann eigenvalue on that half-collar, obtaining a lower bound for $\sigma _1$ depending only on the length of that boundary component. Otherwise, since *f* is $L^2$-orthogonal to 1 on $\partial \Sigma$, it should have a ‘large’ variation somewhere. Either this variation occurs on some $\epsilon$-thin parts adjacent to the boundary, leading to a lower bound for $\sigma _1(\Sigma )$ independent of $\ell _1$, or it occurs away from the boundary. In the latter case, Lemma 2.2 tells us that the variation on the $\epsilon$-thick part is controlled in terms of $\sigma _1$. Thus the ‘large’ variation must happen in the ${\varepsilon _\circ }$-thin parts in the interior, composed of collars around short geodesics. Lemma 2.1 then relates the Dirichlet energy and the length of these short geodesics which ultimately gives the link between $\sigma _1(\Sigma )$ and $\ell _1$. We now proceed with the details of the proof.

### Proof of Proposition 3.3

Let ${{\,\mathrm{\mathscr {C}}\,}}^+_1,\ldots ,{{\,\mathrm{\mathscr {C}}\,}}^+_b$ denote the half-collars around $B_1,\ldots ,B_b$, and ${{\,\textrm{w}\,}}_1,\dots ,{{\,\textrm{w}\,}}_b$ be their corresponding width (${{\,\textrm{w}\,}}_i={{\,\textrm{w}\,}}(B_i))$. For each ${{\,\mathrm{\mathscr {C}}\,}}^+_j$ we consider the Fermi coordinates (*t*, *s*) based on $B_j$, where $0\le t\le {{\,\textrm{w}\,}}_j$ and $0\le s\le |B_j|$. The Riemannian metric on ${{\,\mathrm{\mathscr {C}}\,}}_j^+$ in these coordinates is given by $\, \textrm{d}t^2+\cosh ^2(t)\, \textrm{d}s^2$.

Let *f* be an eigenfunction associated to the first non-zero Steklov eigenvalue $\sigma _1(\Sigma )$ such that $\int _{\partial \Sigma }f^2=1$. We denote by ${\bar{f}}_j$ the average value of *f* over $B_j$ and by ${\bar{f}}_j({{\,\textrm{w}\,}}_j)$ the average of $f({{\,\textrm{w}\,}}_j,\cdot )$ over $B_j$:

$$ {\bar{f}}_j=\frac{1}{|B_j|}\int _{B_j}f(0,s)\, \textrm{d}s,\quad {\bar{f}}_j({{\,\textrm{w}\,}}_j)=\frac{1}{|B_j|}\int _{B_j}f({{\,\textrm{w}\,}}_j,s)\, \textrm{d}s. $$

Since $\int _{\partial \Sigma }f^2=1$, there exists a boundary component $B_I$ such that $\int _{B_I}f^2 \ge 1/b$. Without loss of generality, we assume ${\bar{f}}_I\ge 0$. Note that ${\bar{f}}_I\le \frac{1}{\sqrt{|B_I|}}\Vert f\Vert _{L^2(B_I)}$. We now prove the result considering two separate cases: when $0\le {\bar{f}}_I<\frac{1}{{\sqrt{2b|B_I|}}}$, and when ${\bar{f}}_I \ge \frac{1}{\sqrt{2b|B_I| }}$. Note that if $b=1$, since ${\bar{f}}_I=0$ the first case is the only one one needs to consider.

**Case 1.** $0\le {\bar{f}}_I<\frac{1}{{\sqrt{2b|B_I|}}}$. We define $\tilde{f}:=f-{\bar{f}}_I$. We note that $\int _{B_I}\tilde{f} =0$, and

$$ \int _{B_I}\tilde{f} ^2= \int _{B_I}(f-{\bar{f}}_I)^2=\int _{B_I}f^2-|B_I|{\bar{f}}_I^2\ge \frac{1}{b}-|B_I|{\bar{f}}_I^2\ge \frac{1}{2b}. $$

Hence, we get

$$\begin{aligned} \sigma _1(\Sigma )=\int _{\Sigma }|\nabla f|^2\ge \int _{{{\,\mathrm{\mathscr {C}}\,}}^+_I}|\nabla f|^2=\int _{{{\,\mathrm{\mathscr {C}}\,}}^+_I}|\nabla \tilde{f}|^2\ge \frac{1}{2b}\frac{\int _{{{\,\mathrm{\mathscr {C}}\,}}^+_I}|\nabla \tilde{f}|^2}{\int _{B_I}\tilde{f}^2}\ge \frac{1}{2b} \sigma _1^N({{\,\mathrm{\mathscr {C}}\,}}^+_I), \end{aligned}$$

where $\sigma _1^N({{\,\mathrm{\mathscr {C}}\,}}^+_I)$ is the first non-zero mixed Steklov-Neumann eigenvalue on ${{\,\mathrm{\mathscr {C}}\,}}^+_I$ with Steklov condition on $B_I$ and Neumann condition on the other boundary component of ${{\,\mathrm{\mathscr {C}}\,}}^+_I$. The last inequality follows from the variational characterization of $\sigma _1^N({{\,\mathrm{\mathscr {C}}\,}}^+_I)$. Steklov eigenvalues of warped products in dimension 2 such as the half-collar ${{\,\mathrm{\mathscr {C}}\,}}^+_I$ are explicitly computable. To obtain the value of $\sigma _1^N({{\,\mathrm{\mathscr {C}}\,}}^+_I)$, we observe that the metric $\, \textrm{d}t^2+\cosh ^2(t)\, \textrm{d}s^2$ is conformal to $\frac{1}{\cosh ^2(t)}\, \textrm{d}t^2+\, \textrm{d}s^2$, that is ${{\,\mathrm{\mathscr {C}}\,}}^+_I$ is conformal to the right cylinder $[0, \arctan (\sinh ({{\,\textrm{w}\,}}))]\times \mathbb {S}^1_{R}$ whose mixed Steklov-Neumann eigenvalues are obtained using the method of separation of variables. Since conformal surfaces with conformal factor equal to one along the boundary are Steklov isospectral, we have

$$ \sigma _1^N({{\,\mathrm{\mathscr {C}}\,}}^+_I)=\frac{2\pi }{|B_I|}\tanh \left( \frac{2\pi }{|B_I|}\arctan \left( \frac{1}{\sinh (\frac{|B_I|}{2})} \right) \right) . $$

Therefore,

$$ \sigma _1(\Sigma )\ge \frac{1}{2b}\sigma _1^N({{\,\mathrm{\mathscr {C}}\,}}^+_I)\ge \frac{c_0}{b|B_I|}\min \left\{ 1,\frac{1}{|B_I|e^{|B_I|}}\right\} \ge \frac{c_0}{b\beta {(1+\beta )} e^{\beta }}, $$

for some positive universal constant $c_0$.

**Case 2.** ${\bar{f}}_I \ge \frac{1}{\sqrt{2b|B_I| }}$. We first show that $\sigma _1$ is bounded below by the absolute value of the difference between ${\bar{f}}_I$ and ${\bar{f}}_I({{\,\textrm{w}\,}}_I)$. We have

10 $$\begin{aligned} \begin{aligned} \sigma _1(\Sigma )&=\int _{\Sigma }|\nabla f|^2\ge \int _{{{\,\mathrm{\mathscr {C}}\,}}^+_I}|\nabla f|^2 {=\int _{B_I}\int _0^{{{\,\textrm{w}\,}}_I}\left( |\partial _t f|^2+\cosh (t)^{-2}|\partial _sf|^2\right) \cosh t \, \textrm{d}t\, \textrm{d}s}\\&\ge \int _{B_I}\int _0^{{{\,\textrm{w}\,}}_I} (\partial _t f)^2(t,s)\cosh (t) \, \textrm{d}t {\, \textrm{d}s}\\&\ge \frac{1}{\int _0^{{{\,\textrm{w}\,}}_I} \frac{1}{\cosh (t)} \, \textrm{d}t}\int _{B_I} \left( \int _0^{{{\,\textrm{w}\,}}_I} \partial _tf \, \textrm{d}t \right) ^2 \\&\ge \frac{2}{\pi } \int _{B_I}(f({{\,\textrm{w}\,}}_I,s)-f(0,s))^2 \, \textrm{d}{s}\\&= \frac{2}{\pi }\Vert f_I({{\,\textrm{w}\,}}_I,\cdot )-f(0,\cdot )\Vert _{L^2(B_I)}^2, \end{aligned} \end{aligned}$$

where the inequality between the second and the third lines is obtained by using Cauchy-Schwarz inequality. On the other hand, we have

11 $$\begin{aligned} \begin{aligned} {|}{\bar{f}}_I-{\bar{f}}_I({{\,\textrm{w}\,}}_I){|}&\le \frac{1}{|B_I|}\Vert f_j({{\,\textrm{w}\,}}_j,\cdot )-f(0,\cdot )\Vert _{L^1(B_I)}\\&\le \frac{1}{\sqrt{|B_I|}}\Vert f_I({{\,\textrm{w}\,}}_I,\cdot )-f(0,\cdot )\Vert _{L^2(B_I)}. \end{aligned} \end{aligned}$$

Combining (10) and (11), we get

12 $$\begin{aligned} {\bar{f}}_I-{\bar{f}}_I({{{\,\textrm{w}\,}}}_I)\le |{\bar{f}}_I-{\bar{f}}_I({{\,\textrm{w}\,}}_I)|\le \sqrt{\frac{\pi }{{2|B_I|}}}\sqrt{\sigma _1(\Sigma )}. \end{aligned}$$

Thus, if ${\bar{f}}_I({{\,\textrm{w}\,}}_I)\le \frac{1}{2}{\bar{f}}_I$, replacing in (12) and using our assumption on ${\bar{f}}_I$, we get

13 $$\begin{aligned} \sigma _1(\Sigma )\ge \frac{1}{4\pi b}. \end{aligned}$$

Let us now consider the case ${\bar{f}}_I({{\,\textrm{w}\,}}_I)>\frac{1}{2}{\bar{f}}_I$. It implies that

$$\begin{aligned} \sup _{s\in {[}0,|B_I|{]}}f({{\,\textrm{w}\,}}_I,s)\ge {\bar{f}}_I({{\,\textrm{w}\,}}_I)\ge \frac{1}{2\sqrt{2b|B_I|}}. \end{aligned}$$

Since $\int _{\partial \Sigma } f=0$, we have $\sum _{j\ne I}\int _{B_j} f=\sum _{j\ne I}|B_j|{\bar{f}}_j=-|B_I|{\bar{f}}_I.$ Thus, there exists a geodesic boundary component $B_J$ such that

14 $$\begin{aligned} |B_J|{\bar{f}}_J\le -\frac{|B_I|{\bar{f}}_I}{b-1}\le -\frac{\sqrt{|B_I|}}{(b-1)\sqrt{2b}}. \end{aligned}$$

Note that inequalities (10)–(12) hold for any *j* and are not specific to $j=I$. In particular, we have

15 $$\begin{aligned} \sqrt{|B_J|}| {\bar{f}}_J({{\,\textrm{w}\,}}_J)-{\bar{f}}_J|\le \sqrt{\frac{\pi \sigma _1(\Sigma )}{2}}.\end{aligned}$$

We also have

16 $$\begin{aligned} \inf _{s\in {[}0,|B_J|{]}} f({{\,\textrm{w}\,}}_J,s)\le {{\bar{f}}_J({{\,\textrm{w}\,}}_J)}. \end{aligned}$$

If $\inf _{s\in {[}0,|B_J|{]}} f({{\,\textrm{w}\,}}_J,s)\ge \frac{1}{4\sqrt{2b}\sqrt{|B_I|}}$, then ${\bar{f}}_J({{\,\textrm{w}\,}}_J)\ge \frac{1}{4\sqrt{2b}\sqrt{|B_I|}}.$ It implies

$$\begin{aligned} \sqrt{|B_J|}( {\bar{f}}_J({{\,\textrm{w}\,}}_J)-{\bar{f}}_J)&\ge \frac{\sqrt{|B_J|}}{4\sqrt{2b}\sqrt{|B_I|}}+ \frac{\sqrt{|B_I|}}{(b-1)\sqrt{2b}\sqrt{|B_J|}}\\&\ge \frac{1}{4(b-1)\sqrt{2b}}\left( \frac{\sqrt{|B_J|}}{\sqrt{|B_I|}}+\frac{\sqrt{|B_I|}}{\sqrt{|B_J|}}\right) \\&\ge \frac{1}{4(b-1)\sqrt{2b}}. \end{aligned}$$

Together with (15), we get

$$\begin{aligned} \sigma _1(\Sigma )\ge \frac{1}{16\pi b(b-1)^2}. \end{aligned}$$

We now assume $\inf _{s\in {[}0,|B_J|{]}} f({{\,\textrm{w}\,}}_J,s)<\frac{1}{4\sqrt{2b}\sqrt{|B_I|}}.$ Then

17 $$\begin{aligned} \sup _{s\in {[}0,|B_I|{]}}f({{\,\textrm{w}\,}}_I,s)-\inf _{s\in {[}0,|B_J|{]}}f({{\,\textrm{w}\,}}_J,s)&\ge \frac{1}{4\sqrt{2b}\sqrt{|B_I|}}\ge \frac{1}{4\sqrt{2b\beta }}. \end{aligned}$$

Let $p_I=({{\,\textrm{w}\,}}_I,s_I)$ and $p_J=({{\,\textrm{w}\,}}_J,s_J)$ (points are represented in the Fermi coordinates based on the corresponding geodesic $B_I$ and $B_J$) be such that

$$\begin{aligned} f(p_I)=\sup _{s\in [0,|B_I|]} f({{\,\textrm{w}\,}}_I,s),\quad \text {and}\quad f(p_J)=\inf _{s\in [0,|B_J|]} f({{\,\textrm{w}\,}}_J,s). \end{aligned}$$

Let us consider the ${\varepsilon _\circ }$-thick–thin decomposition of $\Sigma$ as described in Sect. 2. Note that $p_I, p_J\in \Sigma _{{\scriptscriptstyle {\text {thick}}}}^{\varepsilon _\circ }$. Let

$${\varvec{c}}:[0,1]\rightarrow \Sigma \setminus (\bigcup _{j=1}^b{{\,\mathrm{\mathscr {C}}\,}}_j^+\cup (\bigcup _{j=1}^p {{\,\mathrm{{\mathscr {K}}}\,}}_j))$$

be an arbitrary curve connecting $p_I$ and $p_J$ with ${\varvec{c}}(0)=p_I$, ${\varvec{c}}(1)=p_J$. Moreover, we make the following additional assumptions.

- The interval [0, 1] admits a partition $0=t_0<t_1<t_2<\cdots<t_{n-1}<t_n=1$ such that either ${\varvec{c}}([t_i,t_{i+1}])$ is a subset of a connected component of $\Sigma _{{\scriptscriptstyle {\text {thick}}}}^{\varepsilon _\circ }$, or ${\varvec{c}}((t_i,t_{i+1}))\subset {{\,\mathrm{\mathscr {C}}\,}}(\gamma _j)^\circ \subset \Sigma _{\scriptscriptstyle {\text {thin}}}^{\varepsilon _\circ }$ for some *j* with ${\varvec{c}}(t_{i+1})={\varvec{c}}(t_i)^*\in \partial {{\,\mathrm{\mathscr {C}}\,}}(\gamma _j)$, where ${\varvec{c}}(t_i)^*$ is the reflection of ${\varvec{c}}(t_i)$ with respect to $\gamma _j$.
- Each element of the collection $\{{\varvec{c}}((t_i,t_{i+1}))\}$ belongs to a separate connected components of $\Sigma _{{\scriptscriptstyle {\text {thick}}}}^{\varepsilon _\circ }$ or $\Sigma _{\scriptscriptstyle {\text {thin}}}^{\varepsilon _\circ }$.

The number of connected components of the thick and thin parts is bounded by $c_1|\chi |$, where $c_1$ is a positive universal constant. If ${\varvec{c}}([t_i,t_{i+1}])$ is in a connected component $\Sigma _i$ of $\Sigma _{{\scriptscriptstyle {\text {thick}}}}^{\varepsilon _\circ }$, then by Lemma 2.2

$$\begin{aligned} |f\circ {\varvec{c}}(t_i)-f\circ {\varvec{c}}(t_{i+1})|\le {c_2 e^{\beta /2}\sqrt{|\Sigma _i|\sigma _1(\Sigma )}}, \end{aligned}$$

where $c_2$ is a positive universal constant such that the right-hand side is an upper bound for $c(\beta )$ as given in Lemma 2.2. If there exists a curve ${\varvec{c}}$ as described above such that whenever ${\varvec{c}}([t_i,t_{i+1}])$ is a subset of the thin part, we have

$$|f\circ {\varvec{c}}(t_i)-f\circ {\varvec{c}}(t_{i+1})|\le \frac{1}{8{c_1|\chi |}\sqrt{2b\beta }},$$

then

$$\begin{aligned} f(p_I)-f(p_J)&\le \sum _i|f\circ {\varvec{c}}(t_{i})-f\circ {\varvec{c}}(t_{i+1})| \\&\le c_2 e^{\beta /2}\left( \sum _i \sqrt{|\Sigma _i|}\right) \sqrt{\sigma _1(\Sigma )} + \frac{1}{8\sqrt{2b\beta }} \\&\le c_2 e^{\beta /2}\sqrt{c_1 |\chi | |\Sigma |} \sqrt{\sigma _1(\Sigma )} + \frac{1}{8 \sqrt{2b\beta }} \\&\le c_3 e^{\beta /2} |\chi | \sqrt{\sigma _1(\Sigma )} + \frac{1}{8 \sqrt{2b\beta }}. \end{aligned}$$

Combining it with (17) we get

$$\begin{aligned} {\sigma _1(\Sigma )}\ge {\frac{1}{c_4b\beta e^\beta \chi ^2}}, \end{aligned}$$

and we obtain the result. Here, $c_3$ and $c_4$ are positive universal constants.

If such curve does not exist, it means that for any ${\varvec{c}}$ described above there exists an *i* with ${\varvec{c}}|_{[t_i,t_{i+1}]}$ entirely in ${{\,\mathrm{\mathscr {C}}\,}}(\gamma _j)\subset \Sigma _{\scriptscriptstyle {\text {thin}}}^{\varepsilon _\circ }$ for some *j* such that

18 $$\begin{aligned} |f\circ {\varvec{c}}(t_i)-f\circ {\varvec{c}}(t_{i+1})|> \frac{1}{8{c_1|\chi |}\sqrt{2b\beta }}, \end{aligned}$$

then by Lemma 2.1, we have

$$\begin{aligned} \int _{{{\,\mathrm{\mathscr {C}}\,}}(\gamma _j)}|\nabla f|^2 \ge \frac{1}{2^7{c_1^2\chi ^2}b\beta }|\gamma _j|. \end{aligned}$$

Let ${{\,\mathrm{\mathscr {C}}\,}}_{j_1},\cdots , {{\,\mathrm{\mathscr {C}}\,}}_{j_k}$ collection of such collars. Hence,

$$\begin{aligned} \sigma _1(\Sigma )=\int _\Sigma |\nabla f|^2&\ge \sum _m\int _{{{\,\mathrm{\mathscr {C}}\,}}_{j_m}}|\nabla f|^2\ge \frac{1}{2^7{c_1^2\chi ^2}b\beta }\sum _m|\gamma _{j_m}|. \end{aligned}$$

But $\{\gamma _{j_m}\}$ must divide $\Sigma$ into at least two connected components one containing $B_I$ and the other $B_J$. Otherwise, $p_I$ and $p_J$ can be connected by a curve ${\varvec{c}}$ as described above such that there is no interval in the partition for which (18) holds. It contradicts our assumption. Therefore, it is clear that a subcollection of $\{\gamma _{j_m}\}$ gives us a multi-geodesic in ${{\,\mathrm{\mathscr {C}}\,}}_1$ and we conclude that $\sum _m|\gamma _{j_m}|\ge \ell _1$. In summary, we obtain

19 $$\begin{aligned} \sigma _1(\Sigma )&\ge c_5\,\min \left\{ \frac{1}{b^3},\frac{1}{b\beta (1+\beta ) e^{\beta }},\frac{1}{b\beta e^\beta \chi ^2},\frac{\ell _1}{\chi ^2b\beta }\right\} \\ \nonumber&{\ge \frac{c_5}{b\chi ^2}\,\min \left\{ \frac{1}{(1+\beta )^2 e^{\beta }},\frac{\ell _1}{\beta }\right\} } \end{aligned}$$

where $c_5$ is a positive universal constant. We also observe that when $\beta$ is small enough, the minimum is achieved either by the first or the last term in the right-hand side of (19). $\square$

### Remark 3.4

The proof of Proposition 3.3 shows that $\ell _1$ appears in the lower bound of $\sigma _1$ only if there exists a multi-geodesic $c \in {\mathcal {C}}_1$ such that the length of each closed geodesic in *c* is at most $2{\varepsilon _\circ }$. In particular, we can replace $\ell _1$ with $\ell _1^{\varepsilon _\circ }$ in Proposition 3.3. Here,

$$\ell _k^{\varepsilon _\circ }:=\inf \left\{ |{\varvec{c}}|: {\varvec{c}}\in {\mathcal {C}}_k\cap \Sigma _{\scriptscriptstyle {\text {thin}}}^{\varepsilon _\circ }\right\} .$$

We set $\ell _k^{\varepsilon _\circ }=\infty$ if ${\mathcal {C}}_k\cap \Sigma _{\scriptscriptstyle {\text {thin}}}^{\varepsilon _\circ }=\emptyset$. Note that, by abuse of notation, ${\varvec{c}}\in \Sigma _{\scriptscriptstyle {\text {thin}}}^{\varepsilon _\circ }$ means that its image belongs to $\Sigma _{\scriptscriptstyle {\text {thin}}}^{\varepsilon _\circ }$. When $\ell _k^{\varepsilon _\circ }<\infty$, then $\ell _k^{\varepsilon _\circ }=\ell _k$. It shows that when $\Sigma _{\scriptscriptstyle {\text {thin}}}^{\varepsilon _\circ }=\emptyset$, then the lower bound only depends on $\chi , b$ and $\beta$ and not on $\ell _1$.

The next theorem shows that the result of Proposition 3.3 can be extended to some higher-order Steklov eigenvalues.

### Theorem 3.5

Let $\Sigma$ be a hyperbolic surface of signature (*g*, *b*; *p*). Let

$$\begin{aligned} K = {\left\{ \begin{array}{ll} b - 1 &  \text {if} \; (g \ge 1 \, or \, p \ge 2) \, and \, b \, \ge 1, \\ b - 2 &  \text {if} \; g = 0, p = 1 \, and \, b \, \ge 2, \\ b - 3 &  \text {if} \; g = 0, p = 0 \, and \, b \, \ge 3. \end{array}\right. } \end{aligned}$$

Then there exists a positive universal constant *c* such that

$$ \sigma _k(\Sigma ) \ge \frac{c}{b|\chi |^3} \min \left\{ \frac{1}{(1+\beta )^2e^\beta },\frac{\ell _k}{\beta }\right\} ,\qquad 0<k\le K, $$

and

$$ \sigma _{K+1}(\Sigma ) \ge \frac{c}{b\chi ^2(1+\beta )^2e^\beta }. $$

### Remark 3.6

In order to prove this result, we will use a generalisation of Proposition 3.3 to the first non-zero mixed Steklov-Neumann eigenvalue of a hyperbolic surface $\Sigma$ of signature $(g,b^*;p)$ with Steklov condition on $b<b^*$ geodesic boundary components $\{B_1,\dots ,B_b\}$ and Neumann condition on the remaining boundary components $\{B_{b+1},\dots , B_{b^*}\}$. The result involves the quantity $\ell _1^*:= \inf _{{\varvec{c}}\in {{\mathcal {C}}^*_1}} |{\varvec{c}}|,$ where ${{\mathcal {C}}_1^*}$ denotes the set of multi-geodesics formed by disjoint simple closed geodesics, dividing $\Sigma$ into two connected components, each containing at least one boundary component with Steklov condition. When ${{\mathcal {C}}_1^*=\emptyset }$, we set $\ell _1^*=\infty$. Let $\beta ^*:=\max \{|B_i|, i=1,\dots , b^*\}$. Then $\sigma _1^N(\Sigma )$ has the same lower bound as given in Proposition 3.3 with $\ell _1$ replaced with $\ell _1^*$ and $\beta$ with $\beta ^*$.

20 $$\begin{aligned} \sigma _1^N(\Sigma ) \ge \frac{c}{b\chi ^2} \min \left\{ \frac{1}{(1+\beta ^*)^2e^{\beta ^*}},\frac{\ell ^*_1}{\beta ^*}\right\} . \end{aligned}$$

The proof is exactly the same as the proof of Proposition 3.3. If the maximum length of the boundary components with Neumann condition is smaller than $\max \{\beta ,2{{\,\textrm{arsinh}\,}}(1)\}$, where $\beta =\max \{|B_i|: i=1,\ldots , b\}$, then we can replace $\beta ^*$ with $\beta$, and $\ell _1^*$ with $\ell _1^{*,{\varepsilon _\circ }}$ in (20), taking into account Remark 3.4.

Theorem 3.5 holds if we replace $\ell _k$ with $\ell _k^{\varepsilon _\circ }$ and the statements are equivalent. Hence, we prove it in this case using Remarks 3.4 and 3.6 to simplify the argument.

### Proof of Theorem 3.5

For a given $k\in \{1,\ldots ,K+1\}$, let $1\le s\le k$ be the largest *s* such that $\ell _s^{\varepsilon _\circ }\ne \infty$. If such *s* does not exist then $\ell _1^{\varepsilon _\circ }=\infty$ and the result immediately follows from Proposition 3.3 together with Remark 3.4.

Let us first consider the case when $s=k$. Note that it automatically implies that $k\le K$. We consider a curve ${\varvec{c}}=\gamma _1 \cup \dots \cup \gamma _p{\in {\mathcal {C}}_k\cap \Sigma _{\scriptscriptstyle {\text {thin}}}^{\varepsilon _\circ }}$ with $|{\varvec{c}}|={\ell _k^{\varepsilon _\circ }}$. Since $|{\varvec{c}}|= {\ell _k^{\varepsilon _\circ }}$, one of the *p* components of ${\varvec{c}}$ must be of length $\ge \frac{{\ell _k^{\varepsilon _\circ }}}{p}$; we call it $\gamma _{\max }$. We decompose $\Sigma$ into *k* components $\Sigma _1,\ldots ,\Sigma _k$ containing at least one boundary component by removing from $\Sigma$ all the geodesics of ${\varvec{c}}$ except $\gamma _{\max }$. On each $\Sigma _i$, we consider the mixed Steklov-Neumann problem with Steklov condition on $\Sigma _i\cap \partial \Sigma$ and Neumann condition on $\partial \Sigma _i{\cap \Sigma }$. Since the $\Sigma _i$’s are disjoint, by standard variational argument, we have

$$ \sigma _k(\Sigma )\ge \min \{\sigma _1^N(\Sigma _1),\ldots ,\sigma _1^N(\Sigma _{k})\} $$

Because ${\varvec{c}}\in {\mathcal {C}}_k\cap \Sigma _{\scriptscriptstyle {\text {thin}}}^{\varepsilon _\circ }$, all the boundaries of $\Sigma _i$ with Neumann condition are of length $\le {2{\varepsilon _\circ }}\le 2{{\,\textrm{arsinh}\,}}(1)$. Hence, we have from Remark 3.6 that $\sigma _1^N(\Sigma _i)\ge \frac{c_6}{b\chi ^2}\,\min \left\{ \frac{1}{(1+\beta )^2 e^{\beta }},\frac{{\ell _1^{*,{\varepsilon _\circ }}(\Sigma _i)}}{\beta }\right\}$. Moreover, $\ell _1^{*,{\varepsilon _\circ }}(\Sigma _i)\ge |\gamma _{\max }|\ge \frac{{\ell _k^{\varepsilon _\circ }}}{p}$, because otherwise it would contradict the fact that *c* is minimal. We also have $p\le c_7|\chi |$, for some positive universal constant $c_7$. Therefore,

$$\sigma _k(\Sigma )\ge \frac{c_8}{b|\chi |^3}\,\min \left\{ \frac{{\chi }}{(1+\beta )^2 e^{\beta }},\frac{{\ell _k^{\varepsilon _\circ }}}{\beta }\right\} .$$

We now consider the case when $1\le s<k$ and we show that $\sigma _k(\Sigma )\ge \frac{c_6}{b\chi ^2(1+\beta )^2 e^{\beta }}$. We consider a curve ${\varvec{c}}_{s}\in {\mathcal {C}}_s\cap \Sigma _{\scriptscriptstyle {\text {thin}}}^{\varepsilon _\circ }$ such that ${|{\varvec{c}}_{s}|=\ell _{s}^{\varepsilon _\circ }}$. We decompose $\Sigma$ into $s+1$ components $\Sigma _1,\ldots ,\Sigma _{{s+1}}$ containing at least one boundary component by removing from $\Sigma$ all the geodesics of ${\varvec{c}}_s$. On each $\Sigma _i$, we consider the mixed Steklov-Neumann problem with Steklov condition on $\Sigma _i\cap \partial \Sigma$ and Neumann condition on $\partial \Sigma _i{\cap \Sigma }$. We have

$$ {\sigma _k(\Sigma )\ge \sigma _{s+1}}(\Sigma )\ge \min \{\sigma _1^N(\Sigma _1),\ldots ,\sigma _1^N(\Sigma _{s+1})\}. $$

Again, because all the boundaries of $\Sigma _i$ with Neumann condition are of length $\le {2{\varepsilon _\circ }}\!\!\le \! 2{{\,\textrm{arsinh}\,}}(1)$, we have from Remark 3.6 that $\sigma _1^N(\Sigma _i)\!\!\ge \!\! \frac{c_6}{b\chi ^2}\!\min \left\{ \frac{1}{(1+\beta )^2 e^{\beta }}\!\!\frac{{\ell _1^{*,{\varepsilon _\circ }}}(\Sigma _i)}{\beta }\right\}$. We claim that $\ell _1^{*,{\varepsilon _\circ }}(\Sigma _i)=\infty$ for all *i*. If there exists *I* for which there exist ${\varvec{c}}_I\in {\mathcal {C}}^*_1(\Sigma _I)\cap \Sigma _{\scriptscriptstyle {\text {thin}}}^{\varepsilon _\circ }$, then ${\varvec{c}}_I\cup {\varvec{c}}\in {\mathcal {C}}_{s+1}\cap \Sigma _{\scriptscriptstyle {\text {thin}}}^{\varepsilon _\circ }$ and it contradicts the maximality of *s*. In summary, we obtain

$$ \sigma _k(\Sigma )\ge \frac{c_8}{b|\chi |^3}\,\min \left\{ \frac{{\chi }}{(1+\beta )^2 e^{\beta }},\frac{{\ell _k^{\varepsilon _\circ }}}{\beta }\right\} , \qquad 1\le k\le K, $$

and

$$ \sigma _{K+1}\ge \frac{c_6}{b\chi ^2(1+\beta )^2 e^{\beta }}. $$

$$\square $$

We now give a remark on upper bounds.

### Remark 3.7

For every $k\ge 1$, the bounds of the form $\sigma _k(\Sigma )|\partial \Sigma |\le c(|\chi |+k)$ is known for compact surfaces [21, 26] (see also [9, 19]) and it remains true in the setting of finite volume surfaces. As a result, we get

$$\begin{aligned} \sigma _k\le c_1\frac{|\chi |}{\beta }. \end{aligned}$$

Using a classical comparison argument with the Steklov-Dirichlet eigenvalue on half-collar near the boundary, we get (see [31, Lemma 3]):

$$\sigma _k \le c_2e^\beta ,\qquad 1\le k<b.$$

It has been shown in [31] that if $\ell _k$ is sufficiently small, $\sigma _k\le c_3\frac{\ell _k}{\alpha }$, where $\alpha$ is the minimum length of boundary components. The proof is by constructing appropriate test functions around the collar or half collars and constant elsewhere. More precisely, by [31, Proof of Theorem 3], we have the following upper bound for $1\le k\le K$. Let ${\varvec{c}}\in {\mathcal {C}}_k$ such that $|{\varvec{c}}|=\ell _k$. Let $\Sigma _j$ be the connected components of $\Sigma \setminus {\varvec{c}}$, and let $L_j$ denote the length of ${\Sigma _j}\cap \partial \Sigma$. Then

$$\begin{aligned} \sigma _k\le \max _j\frac{\ell _k}{L_j\arctan \left( \frac{1}{\sinh \left( \frac{\ell _k}{2}\right) }\right) }\le \max _j\frac{1}{L_j}\ell _ke^{\ell _k/2}\le \frac{\ell _ke^{\ell _k/2}}{\alpha },\qquad 1\le k\le K. \end{aligned}$$

Note that if there exists ${\varvec{c}}\in {\mathcal {C}}_k$ with $|{\varvec{c}}|=\ell _k$ such that $L_j$ are comparable to $\beta$ then we can replace $\alpha$ by $\beta$.

In summary, we have

$$\sigma _k\le c_4\min \left\{ {e^{\beta }},{\frac{|\chi |}{\beta }},\frac{\ell _ke^{\ell _k/2}}{\alpha }\right\} , \quad 1\le k<K,\quad \text {and}\quad \sigma _{K+1} \le c_1{\frac{|\chi |}{\beta }}.$$

All the results above can be extended if the curvature assumption is relaxed to a pinched negatively curved surface. We first consider the compact setting.

### Theorem 3.8

Let $(\Sigma ,h)$ be a compact Riemannian surface with geodesic boundary, and assume its Gaussian curvature lies in the interval $[-1, -\kappa ]$, where $\kappa \in (0,1)$. Then, the conclusions of Proposition 3.3, Theorem 3.5, and Remark 3.7 still hold, with $\kappa$ and $\kappa ^{-1/2}$ respectively appearing as a multiplicative factor in the lower and upper bounds.

### Proof

Let $\mathcal {D}\Sigma$ denote the double of $\Sigma$. Note that $\mathcal {D}\Sigma$ is a closed surface equipped with an involution whose fixed points are precisely the geodesic boundary. If we use the normalised Ricci flow to deform its metric conformally to the unique hyperbolic metric in the same conformal class (see [11]), then, because the normalised Ricci flow preserves isometries, the resulting hyperbolic metric ${\bar{h}}$ also admits the same involution symmetry along the boundary points. Hence, each component of $\partial \Sigma$ is geodesic in $(\mathcal {D}\Sigma , {\bar{h}})$ because they are fixed points of an isometry. Therefore, we can view $(\Sigma , {\bar{h}})$ as a subdomain of $(\mathcal {D}\Sigma , {\bar{h}})$, whose boundary is geodesic.

Next, write $h = \rho {\bar{h}}$. Similar to what is done in [34], by a generalisation of the Ahlfors–Schwarz argument (see [35]), we have $1 \le \rho \le \frac{1}{\kappa },$ and therefore,

$$ \sqrt{\kappa } \,\sigma _{k}(\Sigma , {\bar{h}})\le \sigma _k(\Sigma , h) \le \sigma _{k}(\Sigma , {\bar{h}}),\quad \text {and}\quad \ell _k(\Sigma ,{\bar{h}})\le \ell _k((\Sigma ,h)\le \frac{1}{\sqrt{\kappa }}\ell _k(\Sigma ,{\bar{h}}). $$

The statement then follows. $\square$

We end with a remark on the non-compact finite-volume setting.

### Remark 3.9

In the statement of Theorem 3.8, if we remove the compactness assumption and assume that the surface has finite volume, then similar results hold.

Indeed, if *h* is asymptotic to a multiple of a hyperbolic cusp metric on each end of $\Sigma$, we can adapt the above argument and perform the normalised Ricci flow [25] and obtain the same statement as in the compact case. If this is not case, we can adapt the proofs of Proposition 3.3 and Theorem 3.5 to get similar results with bounds depending also on $\kappa$ and the expression in terms of $\beta$ may be slightly different. We give hereafter an idea of how this is done. The ${\varepsilon _\circ }$-thick–thin decomposition defined in Sect. 2 holds when the Gaussian curvature of a Riemannian surface $(\Sigma ,h)$ with geodesic boundary lies in the interval $[-1, -\kappa ]$, where $\kappa \in (0,1)$. The primary difference is that the tubes and cusps are diffeomorphic, rather than isometric, to the warped product sets described above. However, the definition of ${\varepsilon _\circ }$ remains unchanged and independent of $\kappa$ (see [8, Theorem 4.3.2] and [2, §10]).

Furthermore, Lemma 2.1 can be established by comparing the Dirichlet energy of a function on a collar around a simple closed geodesic with that in hyperbolic space. Specifically, on ${{\,\mathrm{\mathscr {C}}\,}}(\gamma )$, the metric *h* can be expressed in Fermi coordinates along $\gamma$ as $h = \, \textrm{d}t^2 + A(t,s)^2 \, \textrm{d}s^2$. By writing the Gaussian curvature in terms of *A* and using the curvature bounds, we obtain two second-order differential inequalities which imply

$$\cosh (\sqrt{\kappa }t)\le |A(t,s)|\le \cosh (t),$$

which allows one to adapt the proof of Dodziuk and Randol [15, Lemma 3].

Lemma 2.2 also holds in this context; for a proof, refer to [1, Lemma 4.6]. The constant coefficient in this lemma depends on the norm of the derivatives of the Gaussian curvature. However, there exists a metric quasi-isometric to the original one with some nice properties, where the norm of the derivative of the curvature is bounded (see [1, Remark 4.7]). Therefore, only in the final step of the proof for Case 2 in Proposition 3.3 we need to switch to the quasi-isometric metric before applying Lemma 2.2. A similar approach is used in [1].
